# PM_2.5_ Cooperative Control with Fuzzy Cost and Fuzzy Coalitions

**DOI:** 10.3390/ijerph16071271

**Published:** 2019-04-09

**Authors:** Zhen Zhou, Meijia Zhang, Xiaohui Yu, Xijun He, Kang Wang, Quan Shao, Jie Wang, Hongxia Sun

**Affiliations:** 1 School of Management, Capital Normal University, Beijing 100089, China; zhouzhen@cnu.edu.cn (Z.Z.); zhangmjia@yeah.net (M.Z.); aaron_cnu@163.com (K.W.); 2 School of Logistics, Beijing Wuzi University, Beijing 101149, China; yuxiaohui@bwu.edu.cn; 3 College of Economics and Management, Beijing University of Technology, Beijing 100124, China; hexijun@bjut.edu.cn; 4 School of Economics and Management Engineering, Beijing University of Civil Engineering and Architecture, Beijing 100044, China; shaoquan@bucea.edu.cn; 5 School of Business, Beijing Industrial and Commercial University, Beijing 100084, China

**Keywords:** PM_2.5_ control, fuzzy cooperative game, interval number, Hukuhara–Shapley value

## Abstract

Haze control cost is hard to value by a crisp number because it is often affected by various factors such as regional uncertain meteorological conditions and topographical features. Furthermore, regions may be involved in different coalitions for haze control with different levels of effort. In this paper, we propose a PM_2.5_ cooperative control model with fuzzy cost and crisp coalitions or fuzzy coalitions based on the uncertain cross-border transmission factor. We focus on the Beijing–Tianjin–Hebei regions of China and obtain the following major findings. In the case of haze control in the Beijing–Tianjin–Hebei regions of China, local governments in the global crisp coalition can achieve their emission reduction targets with the lowest aggregated cost. However, Hebei fails to satisfy its individual rationality if there is no cost sharing. Therefore, the Hukuhara–Shapley value is used to allocate the aggregated cost among these regions so that the grand coalition is stable. However, the Beijing–Tianjin–Hebei regions cannot achieve their emission reduction targets in the global fuzzy coalition without government subsidies.

## 1. Introduction

Air pollution is a global problem. Many countries are suffering from haze, such as Mongolia, Pakistan, Saudi Arabia, Egypt, Iran, and China. Haze control is an urgent and difficult task. “Free riding” can be inevitable in haze control due to the negative externalities of cross-border transmission between regions. So, how to balance the interests of multiple parties with an effective cost-sharing mechanism is a key issue. However, it is hard to model air pollution with crisp values because there are all kinds of uncertain information. The transmission rate is difficult to estimate accurately. For example, 28–36% PM_2.5_ of Beijing was transmitted from non-Beijing area [1]. Hence, the direct cost of haze control and the impact of haze control on a region’s economic development cannot be accurately determined. Furthermore, the information among players can be asymmetric and vague in a cooperative game because the players, in order to achieve a favorable outcome, may conceal key information such as their pollution discharge and management. Moreover, the players also may be involved in different coalitions with different levels of effort at the same time because of their limited economic resources [2]. This may result in fuzzy coalitions with incomplete participation among the players.

Zadeh [3] proposed a fuzzy set is a class of objects with a continuum of grades of membership. And the notions of inclusion, union, intersection, complement, relation, convexity, etc., are extended to such sets. Since then, there have been a great number of applications of fuzzy sets. In the literature on the prediction, monitoring, and control of air pollution, Guo et al. [4] and Shad et al. [5] used fuzzy spatial prediction techniques to determine pollution concentration areas in practical situations where observations are imprecise and vague that improved the prediction accuracy and real-time of the air pollutant concentration prediction. Wang et al. [6] developed a novel early warning system based on fuzzy time series to forecast the major air pollutants considering the large fluctuations in the concentration of pollutants. Souza [7] studied the automation of air pollution monitoring using genetic algorithm, fuzzy logic, and neural networks for data from nuclear techniques analysis of industrial waste. Li et al. [8] built a dynamic evaluation model for the purpose of mastering the future air quality immediately based on the method of fuzzy mathematical synthetic evaluation. Fisher [9] illustrated ways in which concepts from fuzzy set theory may be applied to decision-making in the environmental sciences. Later, Zhen et al. [10] and Chen et al. [11] proposed interval-parameter fuzzy programming mixed integer programming method and energy-environment optimization model based on fuzzy set theory, which provided solutions for more efficient pollution reduction. Centobelli et al. [12] proposed the adoption of fuzzy set theory in the field of supply chain and designed a fuzzy-based decision support system. Fan et al. [13] proposed a Stackelberg game model to investigate the profit changes of two coal-electricity price linkage (CEPL) mechanisms caused by different production strategies of coal mining enterprises and coal-fired power plants when coal prices rise to mitigate the serious conflicts between market-driven coal prices and state-administered electricity prices. Some scholars combine fuzzy set theory with game theory to study public goods, such as water resources management. Armaghan [14] studied the optimal allocation of water resources across river basins from the perspective of fuzzy cooperative games. Two fuzzy cooperative game models were established based on water users’ uncertainty of fuzzy income functions and their fuzzy participation degree of coalitions. The results showed that the global coalition resulted in the highest benefit. Armahan [15] further considered political factors to the net income redistribution of the coalition that indicate that considering political factors can provide a solution that makes all water users more satisfied. Moreover, existing studies [16,17,18] provided valuable suggestions for solving water conflicts by using fuzzy cooperative game theory to the water allocation of China’s South-to-North Water Transfer Project. Water resources and haze are both public goods with externality and non-excludability. Hence, the fuzzy cooperative game model is also applicable to haze control. However, a major difference is that water flow is one-way cross-border transmission while haze transmission is bi-directional. So far, limited studies have applied fuzzy game into haze control. Zhou et al. [19] introduced haze cost with interval number and allocated aggregated cost of global coalition by the interval Shapley value. The coalition is vague while the control cost is certain. Sun [20] established a cooperative game model with fuzzy participation and designed an economic benefit coordination mechanism for haze control. However, research is lacking on air pollution with fuzzy cost and fuzzy coalition. The existing studies on PM_2.5_ emission reduction strategies are based on regional total control principle. This means as long as the total amount of regional pollution is up to an abatement request, it is assumed that all regions meet the standards. Such an assumption can lead to “free riding” by some regions, making the emission reduction inefficient. Therefore, the conclusions, and hence the proposed policies from the existing studies, may be unreliable.

There are three contributions in this paper. First, we model the impact of PM_2.5_ emissions with fuzzy cost and fuzzy coalition. Second, we adopt the concentration control principle and consider the impact of regional transmission factors. Third, we provide practical managerial and policy implications for haze control in China and other countries.

The structure of the paper is as follows. In Section 2, the fuzzy aggregated cost function of PM_2.5_ control is constructed. Section 3 establishes a cooperative game model with fuzzy cost and crisp coalition and a cooperative game model with fuzzy cost and fuzzy coalition. Section 4 conducts a case study of the Beijing–Tianjin–Hebei regions in China. Conclusions and policy insights for intergovernmental cooperation on PM_2.5_ as well as future research directions are presented in Section 5.

## 2. Preliminary Conceptions 

### 2.1. Preliminary of Interval Numbers

**Definition** **1.** *If $\tilde{x}=\left[x^{-},x^{+}\right]=\left\{x|x^{-}\leq x\leq x^{+},x,x^{-},x^{+}\in R\right\}$, it is called a standard binary interval number, or interval number in short, where, $x^{-}$ and $x^{+}$ represent the lower and upper limits of the interval number, respectively.*

**Definition** **2.** *Assume $\tilde{x}$ and $\tilde{y}$ are fuzzy numbers. If there is a fuzzy number $\tilde{z}$ such that $\tilde{y}+\tilde{z}=\tilde{x}$, then the Hukuhara-difference of $\tilde{x}$ and $\tilde{y}$ exists. $\tilde{z}$ is called Hukuhara-difference, which is simply the H-difference, recorded as $\tilde{x}-{}_{H}\tilde{y}$*.

**Operation** **1.** *For any interval numbers $\tilde{x}=\left[x^{-},x^{+}\right]$, $\tilde{y}=\left[y^{-},y^{+}\right]$, N={1,2,⋯,n}, there are the following rules of operation:*

$$\tilde{x}\vee \tilde{y}=[x^{-}\vee y^{-},x^{+}\vee y^{+}];\;\tilde{x}\wedge \tilde{y}=\left[x^{-}\wedge y^{-},x^{+}\wedge y^{+}\right].$$

The basic operation of the interval number is a closed operation.

### 2.2. Symbols

Symbols and descriptions of the paper are in Table 1.

Assuming n regions, the fuzzy reduction ${\tilde{P}}_{ri}$ of PM_2.5_ in region i is:

(1) $${\tilde{P}}_{ri}=\sum_{1\leq r\leq n}{\tilde{\delta }}_{ri}[(O_{i}-P_{i})-(O_{il}-P_{il})]$$

where $O_{i}$ and $P_{i}$ are the production and removal of PM_2.5_ in the region, respectively. $O_{il}$ and $P_{il}$ are the production and removal of last year in the region i, respectively. ${\tilde{\delta }}_{ij}$ is the fuzzy contribution rate to region j by the PM_2.5_ emission of region i. i=j indicates its own local influence. The fuzzy contribution rate of PM_2.5_ from region i to region j is calculated as follows:

(2) $${\tilde{\delta }}_{ij}=\frac{{\tilde{\chi }}_{ij}\cdot \beta_{j}\cdot c_{j}}{\sum_{k=1}^{31}{\tilde{\chi }}_{ik}\cdot \beta_{k}\cdot c_{k}}$$

where ${\tilde{\chi }}_{ij}$ represents the fuzzy interval transmission ratio of the average annual PM_2.5_ concentration of region i to region j. $\beta_{j}$ is the conversion coefficient between the mass and the concentration of the region j PM_2.5_. $c_{j}$ is the annual average concentration of PM_2.5_ of the region j. If there is a conversion relationship between the removal and the concentration of PM_2.5_, and the conversion coefficient is $\beta_{i}$, then the relationship between the fuzzy reduction amount ${\tilde{P}}_{ri}$ of PM_2.5_ and the fuzzy falling concentration ${\tilde{\varepsilon }}_{i}$ is:

(3) $${\tilde{\varepsilon }}_{i}={\tilde{P}}_{ri}\cdot \beta_{i}$$

## 3. PM_2.5_ Cooperative Control Model with Fuzzy Cost and Fuzzy Coalitions

### 3.1. The Uncertain Aggregated Cost Function of PM_2.5_ Control

Since the source of PM_2.5_ is mainly affected by gaseous precursors of SO_4_^2−^ and NO_3_^−^ [21], the control of SO_2_ and NO_X_ instead of PM_2.5_ are studied in the paper. The emission reductions of SO_2_ and NO_X_ are all denoted by the interval numbers due to the fuzzy cross-border transmission rate of PM_2.5_. According to Tan et al. [22], the fuzzy control cost function of SO_2_ is:

(4) $${\tilde{C}}_{iso_{2}}=\theta \cdot W_{i}{}^{\varphi }\cdot {\tilde{P}}_{iso_{2}}{}^{\mu }$$

where ${\tilde{C}}_{iso_{2}}$ is the fuzzy control cost of SO_2_ in the region i, $W_{i}$ is the exhaust emission of the region i, ${\tilde{P}}_{iso_{2}}$ is the fuzzy removal of SO_2_ in the region i, and θ,φ,μ are the parameters to be determined. According to [23], the fuzzy control cost function of NO_X_ can be constructed as follows:

(5) $${\tilde{C}}_{iNO_{x}}=\tilde{\sigma }\cdot {\tilde{P}}_{iNOx}$$

where ${\tilde{C}}_{iNO_{x}}$ is the fuzzy control cost of NO_X_ in region i, $\tilde{\sigma }$ is the unit fuzzy control cost, and ${\tilde{P}}_{iNOx}$ is the fuzzy removal amount of NO_X_ in region i. Therefore, the direct control cost function of PM_2.5_ with uncertain information for region i is:

(6) $${\tilde{C}}_{id}=\theta \cdot W_{i}{}^{\varphi }\cdot {\left({\tilde{P}}_{ri}\cdot \mu_{iSO_{2}}\right)}^{\mu }+\tilde{\sigma }\cdot ({\tilde{P}}_{ri}\cdot \mu_{iNO_{X}})$$

where ${\tilde{C}}_{id}$ is the fuzzy direct control cost of PM_2.5_ for region i. $\mu_{iSO_{2}}$ and $\mu_{iNO_{X}}$ are the conversion coefficients between SO_2_ and NO_X_ removal and concentration for region i, respectively. Haze control also may have a certain degree of negative impact on economic development in the control process. Li et al. [24] derived the impact of environmental regulation on 41 industries from the CGE model. The aggregated cost of environmental regulation is:

(7) $$E_{i}=\sum_{s=1}^{41}\nu_{s}\cdot e_{is}$$

where $\nu_{s}$ is the impact of environmental regulation on industry S, and $e_{is}$ is the annual output value of industry S in region i. The fuzzy economic development impact function of region i is:

(8) $${\tilde{C}}_{ie}=\Delta E_{i}\cdot {\tilde{P}}_{ri}\cdot \tau$$

where $\Delta E_{i}$ represents the economic loss caused by the unit concentration of PM_2.5_ per year in region i, and τ is the time factor. The uncertainty of aggregated control cost is the sum of fuzzy direct control cost and economic development impact cost, so the regional uncertain aggregated cost function is:

(9) $${\tilde{C}}_{i}={\tilde{C}}_{id}+{\tilde{C}}_{ie}$$

### 3.2. Fuzzy Cooperative Game Model with Crisp Coalition 

According to Borkotokey [25], there are many types of uncertainties when forming coalitions. In many cases, players can only make vague judgments about the true value of the coalition. Here players are local governments. Coalition means the group negotiating to strive together to reduce emissions. In a crisp coalition, players participate in a coalition with 100% degree of participation, but in a fuzzy coalition, the degree of participation that means players join in a coalition is between 0% and 100%. The central government has set emission reduction concentrations for each region called Emission Reduction Target (ERT). If each player in the coalition can reach its own ERT within the capability of emission reduction, (10) is held. If not, the players must pay control costs to nearby regions in order to achieve the ERT by cutting down the cross-border transmission emission.

(10) $${\tilde{P}}_{ri}\leq P_{pi}$$

Considering the information uncertainty in the coalition, a fuzzy characteristic function denoted by $\tilde{\omega }\left(S\right)$ represents the value of a coalition. Moreover, the regional emission reduction cannot exceed the emission reduction cap. Therefore, the fuzzy characteristic function of a coalition can be represented as:

(11) $$\tilde{\omega }\left(S\right)=\underset{{\tilde{P}}_{r}{}_{i}}{\min}\sum_{i=1}^{s}{\tilde{C}}_{i},S\subseteq N\;s.t.\left\{ \begin{array}{c} \sum_{1\leq r\leq s}{\tilde{\delta }}_{ri}[(O_{i}-P_{i})-(O_{il}-P_{il})]\geq r_{i}\\ 0\leq {\tilde{P}}_{ri}\leq P_{pi},i=1,2,\cdots ,s \end{array}\right.$$

In the crisp coalition $\left(N,\tilde{\omega }\right)$ with transferable payment, set N={1,2,3,⋯,n} represents the global coalitions of all regions participating in the PM_2.5_ cooperative control. P(N) represents the set of all non-empty subsets of the participant set *N*, i.e., the set of all alliances. S is a subset of *N*, i.e., S⊆P(N). $\tilde{\omega }\left(S\right)$ represents the aggregated cost of coalition S and $\tilde{\omega }\in G\left(N\right)$. The Hukuhara–Shapley function [26] $G\left(N\right)\to {\left(R_{+}^{n}\right)}^{P\left(N\right)}$ is defined as:

(12) φi(ω˜)(S)=∑i∈S⊆Nβ(|S|)⋅[ω˜(S∪{i})−ω˜H(S)],β(|S|)=|S|!(n−|S|−1)!n!

where |S| is the number of players in the coalition S and $\varphi_{i}\left(\tilde{\omega }\right)$ is the benefit distribution value of the coalition players. ω˜(S∪{i})−ω˜H(S) is the added benefit for the coalition after joining the coalition S. The cost sharing satisfies the individual rationality $\varphi_{i}\left(\tilde{\omega }\right)\geq \tilde{\omega }\left(\left\{i\right\}\right)$ as well as the collective rationality $\sum_{i\in N}\varphi_{i}\left(\tilde{\omega }\right)=\tilde{\omega }\left(N\right)$.

### 3.3. Fuzzy Cooperative Game Model with Fuzzy Coalition

In a fuzzy coalition, players may participate in a coalition with less than a 100% degree of participation. In the payment-transferable fuzzy Coalition $\left(N,{\tilde{\omega }}^{\prime }\right)$, Q(N) denotes a subset of all fuzzy coalitions, and fuzzy Coalition C∈Q(N), ${\tilde{\omega }}^{\prime }\in H\left(N\right)$. Let C(i) be the participation of i in the fuzzy coalition C, $M\left(C\right)=\left\{C_{i}|C_{i}\geq 0,i\in N\right\}$; m(C) is denoted as the number of elements in the set M(C); that is, the elements in m(C)=|M(C)|=n. M(C) is arranged in increasing order, i.e., $0=h_{0}<h_{1}<h_{2}<\cdots <h_{m\left(C\right)}$. Sun and Zhang [27] defined the characteristic function of a fuzzy cooperative game with uncertain Choquet integral form as:

(13) $${\tilde{\omega }}^{\prime }\left(C\right)=\sum_{m=1}^{m\left(C\right)}\tilde{\omega }\left({\left[C\right]}_{h_{m}}\right)\cdot \left(h_{m}-h_{m-1}\right)$$

The basic form of the Hukuhara–Shapley function is defined as (13) in Section 3.3. For the fuzzy Coalition, the Hukuhara–Shapley function with the uncertain Choquet integral form [26] $H\left(N\right)\to {\left(R_{+}^{n}\right)}^{Q\left(N\right)}$ is defined as:

(14) $$\psi_{i}\left({\tilde{\omega }}^{\prime }\right)\left(C\right)=\sum_{m=1}^{m\left(C\right)}\varphi_{i}\left({\tilde{\omega }}^{\prime }\right)\left({\left[C\right]}_{h_{m}}\right)\cdot \left(h_{m}-h_{m-1}\right)$$

where $\psi_{i}\left({\tilde{\omega }}^{\prime }\right)\left(C\right)$ is the cost of player i joining the fuzzy coalition with participation $C_{i}$.

## 4. Case Study

### 4.1. Overview of the Beijing–Tianjin–Hebei Regions Air Pollution

Since 2013, haze has frequently occurred in North China, especially in the Beijing–Tianjin–Hebei regions. Air pollution has attracted the attention of the central government and scholars. Fan et al. [28] analyzed several drivers of carbon dioxide emissions using the decomposition analysis method based on input and output(IO-SDA) and provided policy advice for low carbonization in the Beijing-Tianjin-Hebei regions. And the Beijing Environmental Protection Monitoring Center has monitored PM_2.5_ concentration from 2013_._ The central government has set ERTs for Beijing, Tianjin, and Hebei. According to central government request, the goal of Beijing’s 2018 action plan is to continue to strive for a decline in annual average PM_2.5_, and to set this goal also for each district. The average concentration reduction in Beijing in 2018 is about 1 μg/m^3^. In addition, the concentrations abatement of Tianjin and Hebei are 1 μg/m^3^ and 4 μg/m^3^, respectively.

As the central government’s ERT is the concentration abatement, it is necessary to convert the decrease of concentration into removal amount. According to the study of the atmospheric environmental capacity of various pollutants by Xue et al. [29], the concentration of PM_2.5_ that can be accommodated in the environmental capacity of 10^4^ tons in the regions of Beijing, Tianjin, and Hebei should be obtained. That is, the conversion coefficients between the removal and concentration of PM_2.5_ in the Beijing, Tianjin, and Hebei regions are $\beta_{1}=7.44$, $\beta_{2}=5.82$, and $\beta_{3}=0.90$, respectively (for convenience, we use subscript 1, 2, and 3 to represent Beijing, Tianjin, and Hebei, respectively).

The PM_2.5_ fuzzy transmission matrix shown in Table 2 is calculated using (4) and the PM_2.5_ space transportation matrix published by the Ministry of Environmental Protection of China includes data from China’s 31 provinces and regions including Beijing, Tianjin, Hebei, Shanxi, and Shandong. The detailed calculation is shown in Table A1, Table A2, Table A3 and Table A4 of Appendix A.

In this paper, the logarithmic regression model predicts the amount and removal mass of PM_2.5_ in 2015. The detailed calculation is shown in Table A5 and Table A6 of Appendix A. According to Xue et al. [30], the upper limit of PM_2.5_ removal is 95% of PM_2.5_ production. Therefore, the upper limit of PM_2.5_ removal $P_{pi}$ in Beijing, Tianjin, and Hebei should be 15.65 $\times 10^{4}$ tons, 15.78 $\times 10^{4}$ tons, and 93.68 $\times 10^{4}$ tons, respectively. Moreover, the basic PM_2.5_ removal $P_{il}$ (PM_2.5_ emission reductions in 2015) are 13.34 $\times 10^{4}$ tons, 11.37 $\times 10^{4}$ tons, and 62.45 $\times 10^{4}$ tons, respectively.

### 4.2. PM_2.5_ Uncertain Aggregated Control Cost Function

The industrial waste gas emissions, the industrial SO_2_ removal in the Beijing–Tianjin–Hebei regions and the control cost of industrial SO_2_ from 2003 to 2010 in China were selected for calculation. According to (4), the control costs of SO_2_ in three regions can be obtained. The functions for Beijing, Tianjin, and Hebei are ${\tilde{C}}_{1SO_{2}}=22.71{\tilde{P}}_{1SO_{2}}{}^{2.042}$, ${\tilde{C}}_{2SO_{2}}=1.038{\tilde{P}}_{2SO_{2}}{}^{2.327}$, and ${\tilde{C}}_{3SO_{2}}=1582.12{\tilde{P}}_{3SO_{2}}{}^{0.617}$, respectively.

The power industry is the largest source of NO_X_ emission. Liu [31] found that the control cost of a power plant using Selective Catalytic Reduction (SCR) technology NO_X_ is about 1.40–1.61 $/kg. Based on the uncertainty of NO_X_ control cost, the NO_X_ fuzzy control cost functions of the three regions are:

$${\tilde{C}}_{1NO_{X}}=\left[1400,1610\right]{\tilde{P}}_{1NO_{X}};{\tilde{C}}_{2NO_{X}}=\left[1400,1610\right]{\tilde{P}}_{2NO_{X}};{\tilde{C}}_{3NO_{X}}=\left[1400,1610\right]{\tilde{P}}_{3NO_{X}}.$$

Therefore, the fuzzy direct control cost functions of the three regions of Beijing–Tianjin–Hebei can be obtained from Equation (6):

$$\begin{array}{l} {\tilde{C}}_{1d}=106.74{\tilde{P}}_{r1}{}^{2.042}+\left[4955.22,5736.50\right]{\tilde{P}}_{r}{}_{1};\\ {\tilde{C}}_{2d}=19.74{\tilde{P}}_{r2}{}^{2.327}+\left[6103.24,7053.21\right]{\tilde{P}}_{r}{}_{2};\\ {\tilde{C}}_{3d}=3150.68{\tilde{P}}_{r}{{}_{3}}^{0.617}+\left[4494.33,5193.88\right]{\tilde{P}}_{r}{}_{3}. \end{array}$$

According to the output value of various industries of the Beijing–Tianjin–Hebei regions in 2015, the aggregated cost of environmental control can be obtained. From Equation (7), the economic loss caused by reducing 10^4^ tons of PM_2.5_ in each region can be obtained (the time factor is set to the GDP growth rate of each region). Therefore, the influence functions of the Beijing–Tianjin–Hebei regional fuzzy economic development are:

$${\tilde{C}}_{1e}=2167.36{\tilde{P}}_{r1};{\tilde{C}}_{2e}=14045.99{\tilde{P}}_{r2};\;{\tilde{C}}_{3e}=4118.69{\tilde{P}}_{r3}.$$

According to Equation (9), the cost functions of PM_2.5_ uncertain aggregated control in Beijing–Tianjin–Hebei regions are:

$$\begin{array}{l} {\tilde{C}}_{1}=106.74{\tilde{P}}_{r1}{}^{2.042}+\left[4955.22,5736.50\right]{\tilde{P}}_{r1}+2167.36{\tilde{P}}_{r}{}_{1};\\ {\tilde{C}}_{2}=19.74{\tilde{P}}_{r2}{}^{2.327}+\left[6103.24,7053.21\right]{\tilde{P}}_{r2}+14045.99{\tilde{P}}_{r2};\\ {\tilde{C}}_{3}=3150.68{\tilde{P}}_{r3}{}^{0.617}+\left[4494.33,5193.88\right]{\tilde{P}}_{r3}+4118.69{\tilde{P}}_{r3}. \end{array}$$

### 4.3. Results and Analysis

When forming a crisp coalition, the three regions of Beijing, Tianjin, and Hebei join the coalition with 100% participation. The crisp coalitions in this paper have the following forms: Individual control, partial coalitions, and global coalition. The fuzzy characteristic functions of the crisp coalitions are represented by the number of intervals.

When Beijing controls PM_2.5_ individually, Beijing’s emission reduction is ${\tilde{P}}_{r1}$ = [13.41, 13.38]$\times 10^{4}$ tons which is less than its emission reduction capacity cap of $P_{p1}$ = 15.64$\times 10^{4}$ tons. According to Equation (10), Beijing can complete its target by controlling PM_2.5_ individually. The fuzzy characteristic value of Beijing is ω({1})= [11.66, 12.73] billion dollars based on Equation (11). Similarly, according to Equation (11), the value of Tianjin and Hebei can be calculated as ω({2}) = [23.95, 25.46] billion dollars and ω({3}) = [65.66, 75.99] billion dollars, respectively.

When Beijing and Tianjin cooperate, Beijing’s emission reduction is ${\tilde{P}}_{r1}$ = [13.29, 15.64]$\times 10^{4}$ tons and Tianjin’s emission reduction is ${\tilde{P}}_{r2}$ = [11.88, 13.68]$\times 10^{4}$ tons. According to Equation (10), they both can achieve ERTs within their emission reduction capacity. Beijing and Tianjin’s emission reduction capacity caps are $P_{p1}$ = 15.64$\times 10^{4}$ tons and $P_{p2}$ = 15.78$\times 10^{4}$ tons, respectively. The fuzzy characteristic value of partial cooperation between Beijing and Tianjin is ω({1,2})= [38.64, 42.32] billion dollars, and the fuzzy cost of individual control in Hebei is [65.66, 75.99] billion dollars.

When Beijing and Hebei cooperate, both can reach the ERTs. The fuzzy characteristic values of partial cooperation between Beijing and Hebei are ω({1,3})= [77.04, 88.36] billion dollars, and the cost of Tianjin’s individual control is [23.95, 25.46] billion dollars. When Tianjin and Hebei cooperate, they can reach the ERTs as well. The fuzzy characteristic value of Tianjin and Hebei partial cooperation is ω({2,3})= [89.03, 99.79] billion dollars, and Beijing’s control cost is: [11.66, 12.73] billion dollars.

When the Beijing–Tianjin–Hebei regions reach a global coalition, the emission reduction in the three regions is ${\tilde{P}}_{r1}$ = [12.70, 13.10]$\times 10^{4}$ tons, ${\tilde{P}}_{r2}$ = [10.79, 11.31]$\times 10^{4}$ tons, and ${\tilde{P}}_{r3}$ = [71.20, 77.70]$\times 10^{4}$ tons. According to Equation (10), it can be concluded that all three regions can achieve ERTs within their emission reduction capacity. The fuzzy characteristic value of the global coalition is ω({1,2,3})= [100.42, 112.19] billion dollars, of which Beijing’s control cost is [11.37, 11.95] billion dollars, Tianjin’s control cost is [23.27, 23.35] billion dollars, and Hebei’s control cost is [65.70, 76.98] billion dollars.

By calculating the fuzzy characteristic value of various coalitions based on Equation (11), these coalitions’ aggregated costs can be obtained. To determine which coalition is the best based on the principle of cost minimization, the sum of the aggregated costs of Beijing–Tianjin–Hebei regions in each coalition form is computed as Table 3. It can be concluded that the aggregated cost of the Beijing–Tianjin–Hebei regions is the smallest with global coalition cooperation. Hence, the global coalition is the best control method. For the members of the coalition, the control costs of Beijing and Tianjin have declined through cooperative control, but the cost of control in Hebei is higher than that of individual control. Therefore, it is necessary to distribute the control costs fairly to achieve a stable global coalition in the long term. The fuzzy eigenvalue table of the cooperative game of the crisp coalition is summarized as below in Table 4.

The Hukuhara–Shapley value is used to share the aggregated cost of global cooperative control. According to Equation (12), Beijing’s cost allocation by joining the global coalition is:

$$\begin{array}{ll} \varphi_{1}\left(\tilde{\omega }\right)\left(\left\{1,2,3\right\}\right)= & \frac{1}{3}\left[\tilde{\omega }\left(\left\{1,2,3\right\}\right)-\tilde{\omega }\left(\left\{2,3\right\}\right)\right]+\frac{1}{6}\left[\tilde{\omega }\left(\left\{1,2\right\}\right)-\tilde{\omega }\left(\left\{2\right\}\right)\right]+\frac{1}{6}\left[\tilde{\omega }\left(\left\{1,3\right\}\right)-\tilde{\omega }\left(\left\{3\right\}\right)\right]\\ & +\frac{1}{3}\left[\tilde{\omega }\left(\left\{1\right\}\right)-\tilde{\omega }\left(\left\{\phi \right\}\right)\right]=\left[11.98,13.25\right] \end{array}$$

Similarly, Tianjin’s cost allocation in the global coalition is $\varphi_{2}\left(\tilde{\omega }\right)\left(\left\{1,2,3\right\}\right)=$ [24.24, 25.33] billion dollars. Hebei’s cost allocation in the global coalition is $\varphi_{3}\left(\tilde{\omega }\right)\left(\left\{1,2,3\right\}\right)=$ [64.18, 73.61] billion dollars.

The results associated with the fuzzy cooperative game model with crisp coalition are summarized in Table 5. It can be seen that after the Hukuhara–Shapley value distribution, Hebei’s control cost is less than the cost of individual control, so joining the global coalition satisfies its individual rationality. Similarly, joining the global coalition is also the best choice for Tianjin. Geographically, Beijing is only connected to Hebei and Tianjin, so Beijing’s non-local emissions are mainly from Hebei and Tianjin. Although Beijing’s control cost of joining the global coalition is slightly higher than its individual control. However, global cooperation control effectively avoids repeated pollution of haze, and helps with emission reduction in Beijing. Thus, Beijing is willing to join the global coalition. Therefore, the global coalition is the best. The aggregated cost of the global coalition is [100.42, 112.19] billion dollars. The control costs in Beijing, Tianjin, and Hebei are [11.98, 13.25] billion dollars, [24.24, 25.33] billion dollars, and [64.18, 73.61] billion dollars, respectively.

The developed regions usually pay more attention to environmental protection. Therefore, economic development level is a key factor in haze control. Considering the actual economic development level, Beijing joined the coalition with 100% participation, while Tianjin and Hebei could not fully join the coalition. That is, they joined the coalition with a certain degree of participation. Next, the fuzzy cooperative game model with fuzzy coalition will be discussed. We assume the participation results of the Beijing–Tianjin–Hebei regions are presented in Table 6.

According to the characteristic function of fuzzy cooperative game with uncertain Choquet integral form as in (13), the fuzzy characteristic functions of fuzzy coalition are calculated, which are presented in Table 7.

Under the fuzzy coalition, the following results are derived based on Equation (12):

$$\begin{array}{ll} \varphi_{1}\left({\tilde{\omega }}^{\prime }\right)\left(\left\{1,2,3\right\}\right)= & \frac{1}{3}\left[{\tilde{\omega }}^{\prime }\left(\left\{1,2,3\right\}\right)-{\tilde{\omega }}^{\prime }\left(\left\{2,3\right\}\right)\right]+\frac{1}{6}\left[{\tilde{\omega }}^{\prime }\left(\left\{1,2\right\}\right)-{\tilde{\omega }}^{\prime }\left(\left\{2\right\}\right)\right]+\frac{1}{6}\left[\tilde{\omega^{\prime }}\left(\left\{1,3\right\}\right)-{\tilde{\omega }}^{\prime }\left(\left\{3\right\}\right)\right]\\ & +\frac{1}{3}\left[{\tilde{\omega }}^{\prime }\left(\left\{1\right\}\right)-{\tilde{\omega }}^{\prime }\left(\left\{\phi \right\}\right)\right]=\left[12.09,13.40\right] \end{array}$$

Similarly, $\varphi_{2}\left({\tilde{\omega }}^{\prime }\right)\left(\left\{1,2,3\right\}\right)=$ [17.25, 18.17] billion dollars and $\varphi_{3}\left({\tilde{\omega }}^{\prime }\right)\left(\left\{1,2,3\right\}\right)=$ [32.09, 36.81] billion dollars. According to Equation (14), Beijing’s cost allocation in the global fuzzy coalition is:

$$\begin{array}{ll} \psi_{1}\left({\tilde{\omega }}^{\prime }\right)\left(\left\{1,2,3\right\}\right) & =\varphi_{1}\left({\tilde{\omega }}^{\prime }\right)\left(\left\{1,2,3\right\}\right)\cdot s_{1}+\varphi_{1}\left({\tilde{\omega }}^{\prime }\right)\left(\left\{2,3\right\}\right)\cdot \left(s_{2}-s_{1}\right)\\ & +\varphi_{1}\left({\tilde{\omega }}^{\prime }\right)\left(\left\{3\right\}\right)\cdot \left(s_{3}-s_{2}\right)=\left[6.05,6.70\right] \end{array}$$

Similarly, the cost allocations of Tianjin and Hebei to the global fuzzy coalition are $\psi_{2}\left({\tilde{\omega }}^{\prime }\right)\left(\left\{1,2,3\right\}\right)=$ [15.26, 16.37] billion dollars and $\psi_{3}\left({\tilde{\omega }}^{\prime }\right)\left(\left\{1,2,3\right\}\right)=$ [34.11, 39.10] billion dollars, respectively.

The above results show that Hebei has the highest cost of control in the fuzzy coalition, followed by Tianjin, and then Beijing. In the case of fuzzy coalition, with the participation degree of 100%, Beijing needs to bear the control cost of [6.05, 6.70] billion dollars. Tianjin’s control cost under the participation degree of 0.7 is [15.26, 16.37] billion dollars. And Hebei’s control cost under the participation of 0.5 is [34.11, 39.10] billion dollars. Moreover, the minimum control costs of the three regions, Beijing, Tianjin, and Hebei, are [11.98, 13.25] billion dollars, [24.24, 25.33] billion dollars, and [64.18, 73.61] billion dollars, respectively, when each player’s participation is 1 and the central government’s ERTs are met. Comparing to the control costs under the crisp coalition and the fuzzy coalition, none of the players can meet the ERTs set by the central government under the fuzzy coalition. Hence, the central government should support, by providing financial subsidies, for example, local governments to increase the participation degree of haze control.

## 5. Conclusions 

In general, it is a challenging task to evaluate haze control cost of a region using a crisp number. This is because of a variety of information uncertainties and the possible forming of coalitions by regions. Therefore, in this paper, we established a model of PM_2.5_ cooperative control with fuzzy costs and crisp coalitions or fuzzy coalitions. We then applied our model to the haze control of the Beijing–Tianjin–Hebei regions in China.

Specifically, with the central government’s emission reduction targets (ERTs), it was assumed that each region joins the coalition with 100% participation. The central government established a PM_2.5_ control cooperation game model with crisp coalition and fuzzy cost, seeking to minimize the costs of local governments and meets ERTs. By calculating the control costs of the global coalition and the partial coalitions, our results show that the global coalition minimizes the aggregated cost. This satisfies the collective rationalities of local governments participating in the global coalition. However, in this case, the control cost of Hebei fails to satisfy its individual rationality. Therefore, in the paper, the Hukuhara–Shapley value was adopted to allocate the aggregated cost of the global coalition according to the marginal contribution, so that each player is willing to join the global coalition. If the local governments are unable to complete the central government’s ERTs within their emission reduction capability caps, the central government can provide subsidies to increase the participation of local governments. Furthermore, as the cross-regional transmission factor changes over time, local government air pollution control expenditures and central government subsidies should also be adjusted accordingly. 

There are more than one future research directions for this paper. First, this paper focuses on PM_2.5_ control in the Beijing–Tianjin–Hebei regions. Further research could consider the interactions among more regions. In such a scenario, a player can join more than one coalition. Second, future research could apply our proposed model to other settings such as water allocation.

## Figures and Tables

**Table 1:** Symbols and description.

Name	Symbols and Description
N	Global coalition in all regions N=(1,2,⋯,n)
S	Partial coalition in some regions, S⊆N
${\tilde{C}}_{id}$	Region i’s fuzzy direct control cost
${\tilde{C}}_{ie}$	Region i’s impact of fuzzy economic development
${\tilde{C}}_{i}$	Region i’s fuzzy aggregation control cost
${\tilde{\delta }}_{ij}$	Region i’s contribution rate of PM_2.5_ emissions to PM_2.5_ of region j, when i=j, indicates local impact
$P_{i}$	Region i’s PM_2.5_ removal
$O_{i}$	Region i’s PM_2.5_ production
$r_{i}$	Region i’s educed concentration when the specified PM_2.5_ is up to standard
$\varepsilon_{i}$	Region i’s PM_2.5_ actually reduces concentration
$P_{pi}$	Region i’s PM_2.5_ removal capacity cap
$P_{il}$	Region i’s PM_2.5_ fuzzy removal last year
${\tilde{P}}_{iSO_{2}}$	Region i’s SO_2_ fuzzy removal
${\tilde{P}}_{iNO_{X}}$	Region i’s NO_X_ fuzzy removal
${\tilde{\sigma }}_{i}$	Region i’s NO_X_ unit fuzzy removal cost
$\beta_{i}$	Region i’s conversion factor between PM_2.5_ emission and concentration
$W_{i}$	Region i’s annual exhaust emission
$\rho_{i}$	Region’s current PM_2.5_ concentration

**Table 2:** PM_2.5_ emission transfer matrix of the Beijing–Tianjin–Hebei regions to other regions (%).

$\delta_{ij}$	j=1	j=2	j=3	Other Provinces
i=1	[49.55, 55.61]	[2.79, 5.21]	[20.22, 31.46]	[13.78, 21.37]
i=2	[2.67, 3.07]	[42.27, 47.48]	[24.59, 31.69]	[23.40, 24.86]
i=3	[1.86, 2.41]	[2.28, 2.88]	[31.40, 51.32]	[43.31, 44.53]

**Table 3:** The sums of the aggregated costs in various coalition forms (billion dollars).

Coalition Forms	Individual	1–2 Partial Coalition	1–3 Partial Coalition	2–3 Partial Coalition	1–2–3 Global Coalition
The sum of the aggregated costs	[101.56, 114.17]	[104.30, 118.31]	[101.29, 113.81]	[100.70, 112.51]	[100.42, 112.19]

**Table 4:** Fuzzy eigenvalue table of the cooperative game of crisp coalition (billion dollars).

Coalition	Fuzzy Eigenvalue	Coalition	Fuzzy Eigenvalue
$\tilde{\omega }\left(\left\{\varphi \right\}\right)$	0	$\tilde{\omega }\left(\left\{1,2\right\}\right)$	[38.64, 42.32]
$\tilde{\omega }\left(\left\{1\right\}\right)$	[11.66, 12.73]	$\tilde{\omega }\left(\left\{1,3\right\}\right)$	[77.04, 88.36]
$\tilde{\omega }\left(\left\{2\right\}\right)$	[23.95, 25.46]	$\tilde{\omega }\left(\left\{2,3\right\}\right)$	[88.03, 99.79]
$\tilde{\omega }\left(\left\{3\right\}\right)$	[65.66, 75.99]	$\tilde{\omega }\left(\left\{1,2,3\right\}\right)$	[100.42, 112.19]

**Table 5:** Comparison of the aggregated costs in individual and global coalition (billion dollars).

Aggregated Costs		Beijing	Tianjin	Hebei
Individual		[11.66, 12.73]	[23.95, 25.46]	[65.66, 75.99]
Global coalition	Before distribution	[11.37, 11.95]	[23.27, 23.35]	[65.70, 76.98]
	After distribution	[11.98, 13.25]	[24.24, 25.33]	[64.18, 73.61]

**Table 6:** Participation degree of cooperation control in the Beijing–Tianjin–Hebei regions.

Region	Participation	Sort	M(C)	$h_{m}\left(C\right)$
Beijing	1	1	$C_{3}$	$h_{3}$
Tianjin	0.7	2	$C_{2}$	$h_{2}$
Hebei	0.5	3	$C_{1}$	$h_{1}$

**Table 7:** Fuzzy eigenvalue table of cooperative game of fuzzy coalition (billion dollars).

Coalition	Fuzzy Eigenvalue	Coalition	Fuzzy Eigenvalue
${\tilde{\omega }}^{\prime }\left(\left\{\phi \right\}\right)$	0	${\tilde{\omega }}^{\prime }\left(\left\{1,2\right\}\right)$	[30.55, 33.44]
${\tilde{\omega }}^{\prime }\left(\left\{1\right\}\right)$	[11.66, 12.73]	${\tilde{\omega }}^{\prime }\left(\left\{1,3\right\}\right)$	[44.35, 50.54]
${\tilde{\omega }}^{\prime }\left(\left\{2\right\}\right)$	[16.97, 17.82]	${\tilde{\omega }}^{\prime }\left(\left\{2,3\right\}\right)$	[49.37, 54.99]
${\tilde{\omega }}^{\prime }\left(\left\{3\right\}\right)$	[32.83, 37.99]	${\tilde{\omega }}^{\prime }\left(\left\{1,2,3\right\}\right)$	[61.44, 68.38]

## Appendix A

Calculation of relevant parameters in the cost function:

### Appendix A.1. PM_2.5_ Fuzzy Transmission Rate in the Beijing–Tianjin–Hebei Regions

By reviewing the environmental bulletins of the Beijing–Tianjin–Hebei regions over the years, the annual PM_2.5_ concentration is constantly changing. According to the source analysis table of PM_2.5_, the local sources and regional transmission ratios are uncertain. Therefore, PM_2.5_ space transportation of 31 provinces (autonomous regions and municipalities) announced by the Environmental Planning Institute of the Ministry of Environmental Protection in 2010 and 2015 are applied to represent the PM_2.5_ fuzzy transmission rate. The regional transmission matrixes extracted from Beijing, Tianjin, and Hebei are as follows:

**Table A1:** Local PM_2.5_ source ratio matrix in the Beijing–Tianjin–Hebei regions in 2010 (%).

	Sources	Beijing	Tianjin	Hebei	Other Provinces
Provinces					
Beijing		63	4	24	9
Tianjin		6	58	26	10
Hebei		5	6	64	21

**Table A2:** Local PM_2.5_ source ratio matrix in the Beijing–Tianjin–Hebei regions in 2015 (%).

	Sources	Beijing	Tianjin	Hebei	Other Provinces
Provinces					
Beijing		66	4	18	12
Tianjin		3	56	20	21
Hebei		3	4	62	31

Based on the uncertain transmission ratio of PM_2.5_ in the Beijing–Tianjin–Hebei regions, the contribution transmission rate is assumed to be an interval number. The lower limit of the interval is the smaller of the transmission rates of PM_2.5_ in 2010 and 2015, and the upper limit is the larger of the transmission rates of PM_2.5_ in 2010 and 2015, the new PM_2.5_ fuzzy transmission matrix in the Beijing–Tianjin–Hebei regions is obtained:

**Table A3:** Local PM_2.5_ source fuzzy proportional matrix in the Beijing–Tianjin–Hebei regions (%).

	Sources	Beijing	Tianjin	Hebei	Other Provinces
Provinces					
Beijing		[63,66]	[4,4]	[18,24]	[9,12]
Tianjin		[3,6]	[56,58]	[20,26]	[10,21]
Hebei		[3,5]	[4,6]	[62,64]	[21,31]

According to the data released by the Environmental Protection Agency in 2016, the annual average concentrations of PM_2.5_ in Beijing, Tianjin, and Hebei are 73 μg/m^3^, 68 μg/m^3^, and 70 μg/m^3^ respectively.

According to Equation (2), it can be concluded that the PM_2.5_ interval contribution matrix of PM_2.5_ in Beijing, Tianjin, Hebei, and other regions is as follows:

**Table A4:** PM_2.5_ fuzzy emission transfer matrix of the Beijing–Tianjin–Hebei regions and other regions (%).

$\delta_{ij}$	j=1	j=2	j=3	Other Provinces
i=1	[49.55,55.61]	[2.79,5.21]	[20.22,31.46]	[13.78,21.37]
i=2	[2.67,3.07]	[42.27,47.48]	[24.59,31.69]	[23.40,24.86]
i=3	[1.86,2.41]	[2.28,2.88]	[31.40,51.32]	[43.31,44.53]

### Appendix A.2. Determination of PM_2.5_ Removal

In the Beijing–Tianjin–Hebei regions, controlling the emission of SO_2_ is the key issue of restricting PM_2.5_, so the amount of PM_2.5_ can be estimated by the production of SO_2_. However, the removal of SO_2_ is no longer provided in the statistical yearbook since 2010, the regression model was used to analyze and predict the removal rate of SO_2_ from 2003 to 2010, comparing the logarithmic regression model, linear regression model and polynomial regression model, data showed that the logistic regression model had the best regression effect. The logarithmic regression model predicted that the SO_2_ removal rates of Beijing–Tianjin–Hebei in 2015 were 79.85%, 68.43%, and 63.24%, respectively.

According to the study of the environmental capacity of PM_2.5_, SO_2_, and NO_X_ by Xue et al. [19], the corresponding relationship between them is obtained:

**Table A5:** Removal of SO_2_ and NO_X_ corresponding to removal of PM_2.5_ (×10^4^ tons).

Corresponding Pollutant Removal	SO_2_	NO_X_
Beijing	2.15	3.55
Tianjin	3.54	4.37
Hebei	3.05	3.22

From the correspondence in the removal of PM_2.5_, SO_2_ and the production of SO_2_ in 2015, the Beijing–Tianjin–Hebei regions predicted by the log-regression model, the removal of PM_2.5_ can be derived as follows:

**Table A6:** Removal of PM_2.5_ for the Beijing–Tianjin–Hebei regions in 2015 (×10^4^ tons).

Regions	PM_2.5_ Removal	PM_2.5_ Production
Beijing	13.1444	16.4597
Tianjin	11.3683	16.6130
Hebei	62.4445	98.7375
